# The Pathophysiological Role of Microglia in Dynamic Surveillance, Phagocytosis and Structural Remodeling of the Developing CNS

**DOI:** 10.3389/fnmol.2017.00191

**Published:** 2017-06-19

**Authors:** Cataldo Arcuri, Carmen Mecca, Roberta Bianchi, Ileana Giambanco, Rosario Donato

**Affiliations:** Department of Experimental Medicine, Centro Universitario per la Ricerca sulla Genomica Funzionale, Perugia Medical School, University of PerugiaPerugia, Italy

**Keywords:** immunosurveillance, microglia phagocytosis, synaptic pruning, autism spectrum disorders, Nasu-Hakola disease, Rett syndrome

## Abstract

In vertebrates, during an early wave of hematopoiesis in the yolk sac between embryonic day E7.0 and E9.0, cells of mesodermal leaflet addressed to macrophage lineage enter in developing central nervous system (CNS) and originate the developing native microglial cells. Depending on the species, microglial cells represent 5–20% of glial cells resident in adult brain. Here, we briefly discuss some canonical functions of the microglia, i.e., cytokine secretion and functional transition from M1 to M2 phenotype. In addition, we review studies on the non-canonical functions of microglia such as regulation of phagocytosis, synaptic pruning, and sculpting postnatal neural circuits. In this latter context the contribution of microglia to some neurodevelopmental disorders is now well established. Nasu-Hakola (NHD) disease is considered a primary microgliopathy with alterations of the DNAX activation protein 12 (DAP12)-Triggering receptor expressed on myeloid cells 2 (TREM-2) signaling and removal of macromolecules and apoptotic cells followed by secondary microglia activation. In Rett syndrome *Mecp2*^-/-^ microglia shows a substantial impairment of phagocytic ability, although the role of microglia is not yet clear. In a mouse model of Tourette syndrome (TS), microglia abnormalities have also been described, and deficient microglia-mediated neuroprotection is obvious. Here we review the role of microglial cells in neurodevelopmental disorders without inflammation and on the complex role of microglia in developing CNS.

## Introduction

Microglia are the resident innate immune cells of CNS. Upon completion of the blood-brain barrier, microglia become physically restricted to the brain as a long-living, autonomous cell population that retains the ability to divide and self-renew during life without any significant contribution from circulating blood cells (Ajami et al., 2007). In adult CNS, microglia have a complex localization with more microglia found in the gray matter than the white matter (Lawson et al., 1990). Microglia vary considerably in morphology. While white matter microglia show elongated somata and processes preferentially oriented along fibers, microglia in the periventricular structures, a region where the blood-brain barrier is leaky, show compact morphology with few short processes. Contrariwise, gray matter microglia are radially ramified (Lawson et al., 1992).

Disturbances of brain homeostasis can determine rapid and profound changes in microglial morphology, gene expression and function. These events define the so-called “microglial activation” (Streit et al., 2005; Hanisch and Kettenmann, 2007; Colton and Wilcock, 2010; Graeber and Streit, 2010) and include changes in gene expression, reorganization of surface molecules for interaction with extracellular environment and neighboring cells, and release of soluble factors acting as pro- or anti-inflammatory factors. Microglia can also become phagocytic to remove tissue debris and damaged cells. The different functional phases of microglia activation are set out on a molecular, functional and morphological basis, and activated microglia resemble macrophages (Hanisch and Kettenmann, 2007; Colton and Wilcock, 2010). The microglial activation is thus a highly regulated process.

Microglia are continuously active. Indeed, “quiescent” microglia actively scan the brain environment, moving the fine processes without perturbing the fragile neural networks. However, these “resting” cells quickly turn into an activated state following minimum alterations of the brain parenchyma (Town et al., 2005). The activation process should also be put in relation to the regional heterogeneity of microglia. For example, Kitada and Rowitch reported that in gray and white matter, microglia show different expression patterns and this heterogeneity was also highlighted in astrocytes and oligodendrocytes (Kitada and Rowitch, 2006). These differences may affect normal microglial function and induce a different response upon inflammatory stimuli (Hristova et al., 2010). Any change in the microenvironment, e.g., the proximity to blood vessels and/or exposure to neurotransmitters, properties of the blood-brain barrier controlling the microenvironment (Abbott et al., 2010), may determine local adaptations of microglia (Davoust et al., 2008). Moreover, microglial heterogeneity was observed in the aging brain (Sierra et al., 2007; Soreq et al., 2017). Possibly, this specificity pertains to housekeeping activities, i.e., phagocytosis of dead neurons, and/or specific activities in response to microglia activation.

In this context, the hypothesis that specialized cells reside among a regional population is cogent. Indeed, expression levels of triggering receptor expressed on myeloid cells (TREM2) in microglial cells were found to differ not only from region to region but also within each region (Schmid et al., 2009) likely due to the capacity of the microenvironment to induce specific microglial phenotypes. Large-scale single cell RNA sequencing (RNA-seq) has confirmed this regional heterogeneity (Zeisel et al., 2015).

Another issue is the behavior of microglia following activation. Although indistinguishable from resting cells, the post-activated microglia could still bear long-lasting adjustments. Epigenetic mechanisms orchestrating long-lasting adjustments are already known. The experienced cell could then assume different behaviors when subjected to the same stimulus. Conceptually, any given population may develop altered microglial functions and succumb more quickly to the stimulus, or may respond more quickly and effectively upon a second challenge (Conde and Streit, 2006).

## Origin and Localization of Microglia

The existence of microglial cells within the CNS was demonstrated a century ago by del Rio-Hortega who is considered “the father of microglia”. Despite a great deal of information about microglia, many features of these cells are still controversial, including the identity of their precursors. Historically, two main hypotheses of microglial cells’ origin were put forward. The first hypothesis, which was supported by a large fraction of scientists, stated that microglial precursors originate from the mesenchyme, while the second one stated that microglial cells originate from neuroepithelial cells.

del Rio-Hortega supposed that microglial cells arose from the embryonic cells of the meninges, asserting that these cells derived from the mesenchyme. To support his hypothesis, he remarked that these cells showed features similar to those of blood leucocytes and probably were blood-born cells (del Rio-Hortega, 1932). Indeed, microglial cells and cells of the monocytic lineage share the expression of peculiar enzymes such as acid phosphatase, non-specific esterase and nucleoside diphosphatase, the presence of vault particles, and the ability to bind the same lectins (Cuadros and Navascués, 1998). Also, the differentiation of cells of the myeloid lineage fails in mice lacking the transcription factor PU.1 and microglial cells cannot be recognized in the CNS of these mice (McKercher et al., 1996). Nevertheless, a weak point of this hypothesis comes from the finding that macrophages/microglia appear within the CNS before the development of vessels and before the production of monocytes in hematopoietic tissue, leading the possibility that microglia may originate from undifferentiated hematopoietic precursors that autonomously enter the developing CNS (Ashwell, 1991). Consistent with this observation, cells with the ability to differentiate into microglial-like (Mac-1^+^, Mac-3^+^, F4/F80^+^ and Fc-R^+^) cells can be detected in the developing mouse neuroepithelium at days E8.5/E9.0 and others detected amoeboid cells expressing macrophage and/or microglial markers at similar developmental stages (Alliot et al., 1991). The possibility that the yolk sac might be the site of origin of microglial progenitors was supported by the finding that at days E8.5/E9.0 the yolk sac is the only hematopoietic site in the embryo; indeed, macrophage precursors were found in the yolk sac before microglial precursors could enter the neural tube, which led to the conclusion that these cells move from the yolk sac into the mesenchyme of the developing brain initially by interstitial migration and later on through the blood circulation, after which they become established in the neuroepithelium (Alliot et al., 1999). Similar findings were reported in humans; starting at 4.5 weeks of gestation, amoeboid microglial cells expressing ionized calcium-binding adapter molecule (Iba)-1, Cluster of Differentiation (CD) 68, CD45 and Major Histocompatibility (MHC)-II enter the cerebral wall from the ventricular lumen and the leptomeninges (Monier et al., 2007). Further studies confirmed that the origin of microglial cells from yolk sac progenitors is conserved across some species, such as zebrafish and avians; the difference between these species and mice is represented by the requirement, in the latter ones, of a functional blood circulation for an adequate distribution of macrophages in the embryo (Herbomel et al., 1999, 2001).

To date, it is widely accepted that microglial cell precursors develop in the yolk sac and Ginhoux et al. (2010) additionally demonstrated that, postnatal hematopoietic progenitors are not involved in the maintenance of adult microglial cells. Furthermore, these same authors highlighted the fact that, differently from circulating monocytes and macrophages, the development of microglial cells is strictly dependent on colony stimulating factor-1 receptor (CSF-1R) and that microglial cells develop in *Csf-1^op/op^* mice because during early brain postnatal development, the mRNA expression level of IL-34, which is another CSF-1R ligand, is higher than that of CSF (Ginhoux et al., 2010). Currently, there is evidence that in mice non-parenchymal (perivascular, meningeal and choroid plexus) macrophages arise in the yolk sac and persist during life without replacement from bone marrow with the exception of choroid plexus macrophages (Goldmann et al., 2016).

However, the identity of microglial precursors and their gene expression profile still are an unresolved issue. Recently, it was demonstrated that c-kit^+^ erythromyeloid precursors (EMPs) are present in the yolk sac at day 8 post conception and that EMPs become first CD45^+^, c-kit^lo^, chemokine C-X3-C motif receptor 1 (CX3CR1)^-^ (A1) cells and then differentiate into CD45^+^, c-kit^-^ CX3CR1^+^ (A2) mature cells, showing an up-regulated expression of F4/80 and of macrophage CSF receptor (Kierdorf et al., 2013). Moreover, the same authors found that the transcription factors interferon regulatory factor 8 and Pu.1 are necessary for the proper development of A1 and A2 precursors but not of EMPs, whereas other factors such as kruppel-like factor 4, inhibitor of DNA binding 2, cellular myeloblastosis transcription factor and basic leucine zipper transcription factor ATF-like 3, are not required. They also showed that, differently from the injured brain, microgliogenesis is independent of the chemochine C-C motif ligand 2 (CCL2) and the chemokine receptor CCR2 and is instead regulated by the activity of metalloproteases 8 and 9 (Kierdorf et al., 2013).

Like microglial cell precursors, tissue resident macrophages such as kidney macrophages, osteoclasts, Kupffer cells, alveolar macrophages and Langerhans cells arise in the yolk sac, develop without the contribution of c-Myb, which is instead necessary for hematopoietic stem cell (HSC) development and are maintained as a stable population during adulthood. These evidences suggest that, as observed in microglial cells, the development of tissue macrophages is independent of HSCs (Schulz et al., 2012). Furthermore, Gomez Perdiguero and colleagues proved that mouse Kupffer cells, microglial cells, Langerhans cells and alveolar macrophages originate from *Tie2*^+^ EMPs that arise in the yolk sac and colonize the liver before E10.5 where they develop into fetal macrophages, granulocytes, monocytes and erythrocytes. After E16.5, while fetal erythrocytes, granulocytes and monocytes are replaced by HSC-derived cells, Kupffer cells, microglial cells and Langerhans cells persist as quite stable populations; instead, lung alveolar macrophages and gut associated-macrophages can be replaced in older mice (Gomez Perdiguero et al., 2015).

Another issue that needs to be elucidated is the contribution of circulating monocytes to the adult microglial population in physiological and pathological conditions; microglia participate in the pathophysiology of many diseases including Alzheimer’s disease (AD) and Parkinson’s disease (Kokovay and Cunningham, 2005; Malm et al., 2005). There is evidence that, after injury or irradiation, monocytic (Ly-6C^hi^Gr-1^+^CCR2^+^CX3CR1^lo^) cells are recruited from the peripheral blood to the CNS where they differentiate into microglial cells (Mildner et al., 2007). Thus, microglia are brain resident macrophages, but their population can increase in pathological conditions via recruitment of monocytes from the bloodstream.

## Molecular Markers (Phenotypes)

**FIGURE 1:**
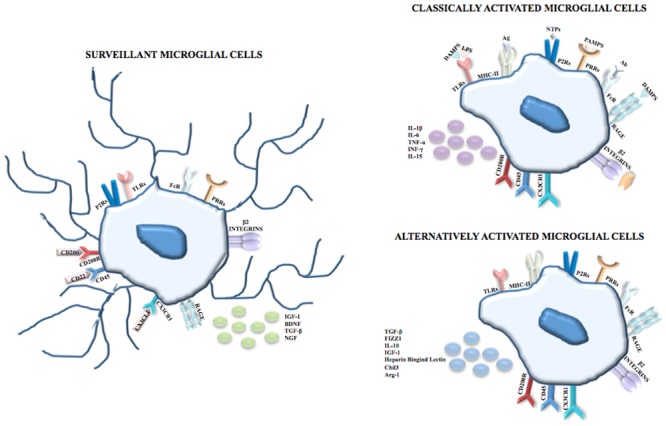
Microglial cells. In physiological conditions and during CNS development, microglia are surveillant cells showing a ramified morphology and, according to the monocytic/macrophage lineage, express on their membrane surface β2-integrins (CD11a, CD11b), Fc receptors, and PRRs including TLRs, and P2Rs and RAGE. The surveillant state is maintained through inhibitor signals mediated by the interactions between CD200 and CD200R, CD22 and CD45, and between CX3CL1 and CX3CR1 and is characterized by the release of IGF-1, BDNF, TGF-β and NGF. Following recognition of PAMPs such as LPS, viral envelops, bacterial cell wall components, the DAMPs nuclear protein HMGB1 and S100B, NTPs or complement proteins, microglia shift to the classical activated state (known as M1 phenotype) which is characterized by the upregulation of the MHC class II and by the release of pro-inflammatory cytokines, i.e., IL-1β, IL-6, TNF-α, INF-γ and IL-15. However, in order to limit neuronal damage, the inflammation needs to be tightly regulated. Thus, after the induction of the immune response, microglial cells shift to the alternative activated state (knows as M2 phenotype) which is characterized by the induction of Arginase 1 (that promotes wound healing), heparin-binding lectin, and chitinase 3 (that prevents the degradation of extracellular matrix found in inflammatory zone and favors the deposition of extracellular matrix), and by the release of the anti-inflammatory cytokines IL-10, TGF-β and of the growth factor IGF-1.

Making an exhaustive list of microglial cell molecular markers is challenging for two main reasons. Firstly, microglia share the expression of some antigens with other immune cells (e.g., macrophages and lymphocytes) and with endothelial cells and oligodendrocytes (Guillemin and Brew, 2004). Secondly, the literature concerning this topic is often contradictory because of numerous variables including interspecies differences, culture conditions, activation and ability to proliferate (Guillemin and Brew, 2004). However, among the markers of the monocytic/macrophage lineage, microglial cells express β2-integrins (CD11a, CD11b) whose ligands are Intercellular Adhesion Molecule 1 for CD11a and complement proteins for CD11b; LCA which is a family of tyrosine protein phosphatase receptors involved in signal transduction; and receptors for the fragment crystallizable (Fc) chain of immunoglobulins. Reactive microglial cells express MHC class I antigens and some of these cells concomitantly express both MHC class I and class II antigens and interact with T-helper/inducer (T4) and T-cytotoxic/suppressor (T8) classes of lymphocytes (Kim and de Vellis, 2005). In physiological conditions microglia exhibit an expression profile characterized morphologically by the presence of branched processes that continuously monitor the surrounding parenchyma; it was demonstrated that surveillant microglia are able to produce and secrete neurotrophic factors, including insulin-like growth factor 1 (IGF-1), brain-derived neurotrophic factor, transforming growth factor (TGF)-β and nerve growth factor (NGF) (**Figure 1**).

Following the recognition of infection or injury, microglia rapidly shift to an activated state (known as M1 phenotype) that is characterized by the expression of MHC class II antigens and by the production of pro-inflammatory cytokines. The identification of pathogen-associated molecular patterns (PAMPs) occurs because microglia express on their membrane surface pattern recognition receptors, including toll-like receptors (TLRs), RIG-I like receptors, NOD-like receptors, C-type lectin receptors and the receptor for advanced glycation end-products (RAGE). Among TLRs, TLR4 is crucial for the recognition of lipopolysaccharide (LPS), a cell component of Gram-negative bacteria. TLRs are also able to identify endogenous damage-associated molecular patters (DAMPs) that are induced by metabolic products, such as oxidized low density lipoproteins, and/or are released by damaged/dead cells. Additionally, DAMPs are recognized by microglia through ion channels and neurotransmitter receptors that contribute to clear the brain parenchyma from cell debris and help to begin tissue repair after injury. Many P2 purinoreceptors are expressed on microglia and this family comprises ionotropic receptors (P2X), opened by the binding of adenosine triphosphate (ATP) and metabotropic receptors (P2Y) that bind purines or pyrimidines, resulting in the activation of downstream pathways through the activation of G proteins. Recently, *P2ry12* encoding purinergic receptor P2RY12 was found expressed in microglial cells but not myeloid cells of spleen, bone marrow and peripheral blood (Butovsky et al., 2014). Also, P2RY12^+^ cells co-localize with green fluorescent protein (GFP) that labels microglia in *CX3CR1^GFP^* mice but not with GFAP^+^ astrocytes or NeuN^+^ neurons. The exclusive expression of P2RY12 in microglia was independently confirmed through direct RNA-seq and dual fluorescent *in situ* hybridization analysis in which microglial and macrophage transcripts of the same mice were compared in order to find gene expression similarities and differences (Hickman et al., 2013). The same analysis also revealed that the expression of the enzyme Hexosaminidase B in the brain is restricted to microglia.

Nucleoside triphosphates (NTPs) are released by injured cells and, after the binding to P2X or P2Y receptors, trigger the extracellular signal regulated kinase (ERK) pathway that in turn activates the transcription factors nuclear factor kappa-light-chain-enhancer of activated B cells (NF-κB) and activator protein 1, thereby inducing the transcription of pro-inflammatory mediators. Also, RAGE can be activated by DAMPs such as the nuclear protein high mobility group box 1 (HMGB1) released by necrotic cells with resultant activation of the transcription of pro-inflammatory genes and the calcium binding protein S100B released in high amounts by astrocytes following astrocyte damage and necrosis. At high extracellular levels S100B upregulates the expression of the pro-inflammatory enzyme, cyclo-oxygenase 2 (Sorci et al., 2010; Saijo and Glass, 2011) (**Figure 1**).

However, the pro-inflammatory response needs to be tightly regulated in order to limit neuronal damage; for this reason, after the induction of the immune response, microglia shift to the alternative activated phenotype (David and Kroner, 2011; Boche et al., 2013; Hu et al., 2015) known as M2, that is further divided into M2a-M2c subtypes (Chhor et al., 2013). Besides limiting the destructive immune response, the M2 phenotype promotes wound healing through high levels of arginase-1 (Arg-1) (Fenn et al., 2014). Arg-1 converts arginine to ornithine for wound healing (Campbell et al., 2013) and at the same time, by utilizing arginine which is the same substrate as that of the inducible nitric oxide (NO) synthase (iNOS), Arg-1 competes with iNOS thus reducing the production of NO (Pesce et al., 2009; Campbell et al., 2013). In the context of neuroprotection, the M2 phenotype induces chitinase-like 3 (also known as Ym1) (Pepe et al., 2014), a heparin-binding lectin that prevents the degradation of extracellular matrix and FIZZ 1 (found in inflammatory zone 1) that promotes the deposition of extracellular matrix (Pepe et al., 2014) as well as anti-inflammatory cytokines and growth factors (IL-10, TGF-β, IGF-1) (Arcuri et al., 2017) (**Figure 1**).

Although the terms M1 and M2 are widely used to define classically and alternatively activated microglia, the opportunity to use this terminology, originally referred to tissue macrophages, is debated (Ransohoff, 2016). Indeed, transcriptomic, proteomic, two photon microscopy and epigenomic studies show increasing differences between microglia and macrophages in terms of genesis, survival, ultrastructural characteristics, gene expression profile, functions and responses to injury, highlighting the need to change our way to consider and classify microglia (Ransohoff, 2016).

## Mechanisms of Action of Microglia

### Phagocytosis

Microglia are the professional phagocytes of the brain and this activity relies on “eat-me” and “don’t-eat-me” signals. In general, two types of receptors come into play in microglial phagocytosis, that is TLRs which bind with a high affinity to microbial pathogens, and TREM2 which recognizes apoptotic debris. Still other receptors participate in microglial clearance of apoptotic cells and dead or dying neurons (Aderem and Underhill, 1999; Diaz-Aparicio et al., 2016).

**FIGURE 2:**
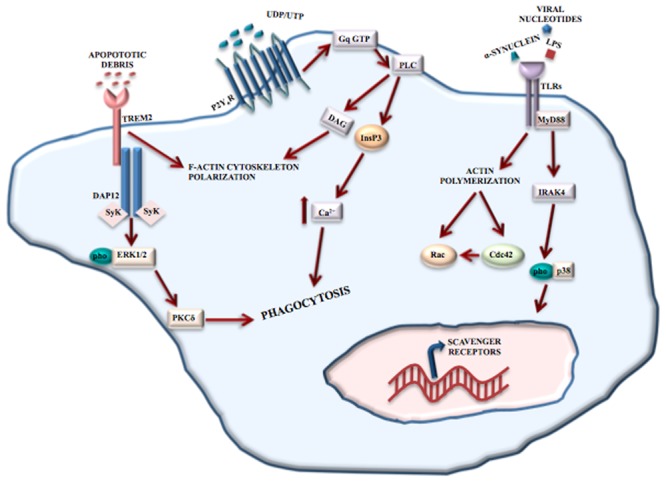
Phagocytosis mediated by receptors. Microglial phagocytosis needs principally two type receptors: TREM2 and TLRs. TREM2 recognizes apoptotic cell debris and, through the binding to DAP12, induces F-actin reorganization and ERK phosphorylation, resulting in apoptotic neuron clearance. TLRs recognize structurally conserved molecules of microbes, DAMPs and α-synuclein. The activation of TLRs results in the induction of the MyD88-dependent pathway that, through IRAK-4 and p38 phosphorylation, induces the upregulation of scavenger receptors. TLRs also contribute to phagocytosis through a MyD88-independent actin-Cdc42/Rac pathway. However, P2Y6 receptor also contributes to UDP-evoked microglial phagocytosis through the activation of phospholipase C (PLC) that in turn triggers InsP3 synthesis-dependent Ca^2+^ release from InsP3-receptor-sensitive stores. In addition, P2Y6-receptor-dependent signaling promotes actin cytoskeleton polarization facilitating the engulfment of cell debris.

Toll-like receptors recognize structurally conserved molecules of microbial origin. TLRs 1-9, belonging to the 3 superfamily, are expressed exclusively on antigen presenting cells including microglia (Collin et al., 2013), macrophages, antigen presenting dendritic cells, and neural cells. TLRs also recognize DAMPs, such as α-synuclein and Aβ fibril (Hanke and Kielian, 2011) and are implicated in many different physiological processes such as learning, memory and neurogenesis (Hanke and Kielian, 2011). A variety of cerebral diseases, including neurodegenerative and inflammatory demyelinating disorders, viral and bacterial infections involve TLRs’ functions (Hanke and Kielian, 2011). TLR2, TLR4, and TLR9-dependent signaling pathways have a protective role in nerve regeneration (Caso et al., 2008; Lee and Landreth, 2010) and affect microglial phagocytosis of neurotoxic Aβ deposit in the AD brain. TLR2 and TLR4 are involved in mediating brain injury and the consequent inflammation after ischemic stroke (Rivest, 2003; Caso et al., 2008). TLRs regulate phagocytosis via myeloid differentiation factor 88 (MyD88)-dependent (Doyle et al., 2004) and MyD88-independent (Kong and Ge, 2008) signaling pathways (**Figure 2**).

TREM2 is a receptor located mainly at the cell surface of osteoclasts and in microglia (Klesney-Tait et al., 2006). Activation of TREM2 receptors mediate protective phagocytosis of apoptotic cell debris, suppress secretion of pro-inflammatory factors such as reactive oxygen species and cytokines and upregulate the synthesis of chemokines (Klesney-Tait et al., 2006). Clinical observations have shown the involvement of TREM2 in Nasu-Hakola disease (see Role of Microglia in Neurodevelopmental Disease), and recently, TREM2 mutations have been associated with an increased risk of developing amyotrophic lateral sclerosis (Cady et al., 2014), frontotemporal dementia (FTD) (Thelen et al., 2014) and AD (Jonsson et al., 2013). Although a complete picture of the signaling pathways and/or intracellular mediators is missing, TREM2 engagement in microglia leads to the reorganization of F-actin and phosphorylation of ERK1/2, by binding to DNAX activation protein 12 (DAP12), thereby mediating the clearance of apoptotic neurons (Piccio et al., 2007) (**Figure 2**).

P2Y6 receptor is responsive to uridine diphosphate (UDP) and partially to uridine triphosphate and adenosine diphosphate. P2Y6 receptor engagement results in UDP-evoked microglial phagocytosis (Koizumi et al., 2007). Released from injured neurons, UDP essentially acts as an “eat-me” signal and mediates the P2Y6-induced phagocytosis. By combining with UDP, P2Y6 activates phospholipase C with resultant synthesis of inositol 1,4,5-trisphophate (InsP3) and release of Ca^2+^ from InsP3-receptor-sensitive stores (Koizumi et al., 2007). In addition, P2Y6-receptor-dependent signaling determines actin reorganization to induce filopodia-like projections, thus facilitating the engulfment of cell debris (Koizumi et al., 2007) (**Figure 2**).

In the CNS, phagocytosis is mediated by receptors as well as by exposure of “eat-me” and “don’t-eat-me” signals. The prevalence of one signal or the other one determines the occurrence or non-occurrence of phagocytosis. The exposure of phosphatidylserine (PS) on neurons is the most important “eat-me” signal (Elward and Gasque, 2003; Zhu et al., 2012) but its exposure is not itself harmful to neurons. PS becomes exposed contingent on ATP depletion or increased calcium levels (Suzuki et al., 2013a). Moreover, PS translocases and phospholipid scramblases have an important role (Suzuki et al., 2013b) with the former ones being inhibited by oxidative stress. The 4 P-type ATPases, ATP8A1 and ATP8A2 translocases, constantly pump PS in the inner leaflet of plasma membrane and ATP8A2 null mutations determine neurodegeneration in mice (Levano et al., 2012; Zhu et al., 2012). Yet, the molecular details of neuronal exposure of PS are unclear. However, it is remarkable that PS exposure is not an irreversible event. In stressed neurons, growth factor withdrawal (Kim et al., 2010) and oxidative stress (Neher et al., 2013) might contribute to reversible PS exposure.

**FIGURE 3:**
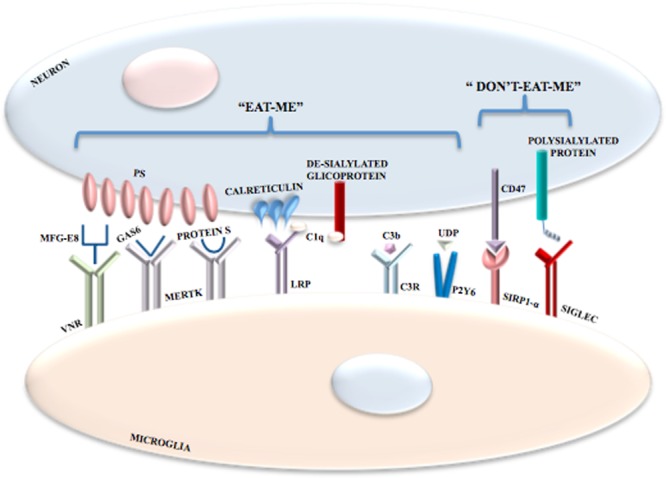
Phagocytosis mediated by the exposure of “eat-me” and “don’t-eat-me” signals. In addition to receptor activation, microglial phagocytosis is also triggered by the exposure of “eat-me” and “don’t-eat-me” signals. Among “eat-me” signals there is the exposure of PS on neurons following oxidative stress, increase in Ca^2+^ levels or ATP depletion; PS binds VNRs through the interaction with MFG-E8. PS also binds MERTK through the interaction with both GAS6 and Protein S. Calreticulin is another neuronal “eat-me” signal that binds LRPs on microglial cells. Also the complement components C1q and Cb3 induce phagocytosis; C1q binds de-sialylated neuronal glicoproteins and is recognized by LRPs in association with calreticulin, whereas C3b binds C3R. Microglial phagocytosis is inhibited by inhibitory signals referred to as “don’t-eat-me” signals mediated by the interaction between CD47 and SIRPα and between polysialylated proteins and SIGLECs.

Several microglial receptor may participate in phagocytosis. Milk fat globule EGF 8 (MFG-E8) (Fricker et al., 2012a; Neniskyte and Brown, 2013) is secreted by microglia and links exposed neuronal PS to microglial vitronectin receptors (VNRs) (Hanayama et al., 2002; Fricker et al., 2012a). Activated VNRs stimulate phagocytosis via remodeling of the microglial actin cytoskeleton (**Figure 3**). Microglial MER receptor tyrosine kinase (MERTK) also mediates neuronal phagocytosis (Wu et al., 2005; Grommes et al., 2008) as does microglial formyl peptide receptor 2. This latter receptor is activated by the interaction between microglia-secreted annexin 1 and neuronal PS (McArthur et al., 2010). It is also important to distinguish between non-inflammatory and inflammatory conditions and between receptors that mediate phagocytosis indirectly or directly. For example, in non-inflammatory conditions brain-specific angiogenesis inhibitor 1 may be a direct receptor for PS, if exposed, probably mediating microglial phagocytosis of axons of live neurons (Marker et al., 2012). When the inflammation processes is started, specific PS-binding opsonins and their receptors are expressed enabling efficient identification, with resultant phagocytosis, of PS-exposing cells. Indeed, MERTK is upregulated in microglia by brain inflammation (Neher et al., 2013). Is quite clear that several opsonins and phagocytic receptors shall be utilized in specific situations, probably, but not necessarily, to remove specific neuronal structures. Moreover, some receptors (e.g., VNRs) mediate phagocytosis directly whereas others (e.g., TLRs) increase phagocytosis indirectly by, for example overexpressing opsonins and phagocytic receptors, with some receptors mediating phagocytosis both indirectly and directly, though.

Microglia and astrocytes produce the complement components C1q and C3 that bind to altered neuronal surfaces inducing phagocytosis. De-sialylated neuronal cell surface glycoproteins are recognized by C1q, whereas C3, after conversion to C3b and through complement receptor 3, opsonizes neurons (Schafer et al., 2012; Linnartz et al., 2012) (**Figure 3**). Moreover, damaged neurons may locally release UDP which recruits P2Y6 receptors thereby inducing phagocytosis (Koizumi et al., 2007) (**Figure 3**).

Calreticulin is another “eat-me” signal in neurons. Exposure of calreticulin promotes the phagocytosis of neurons by binding to microglial low-density lipoprotein receptor-related protein (LRP) (Fricker et al., 2012b) (**Figure 3**).

Phagocytosis of neurons by microglia can also be modulated by inhibitory signals. Polysialylated proteins on the neuronal surface inhibit phagocytosis via activation of members of the sialic acid-binding immunoglubulin-like lectins (SIGLECs), including SIGLEC-E (in mice) and SIGLEC-11 (in humans) on the surface of microglia (Wang and Neumann, 2010; Claude et al., 2013) (**Figure 3**). Expression of CD47 on cells and myelin inhibit microglial phagocytosis via binding to signal regulatory protein-α, the CD47 receptor (Gitik et al., 2011). However, whether CD47 is a major “don’t-eat-me” signal in neurons is unclear. Plasminogen activator inhibitor type 1 is a “don’t-eat-me” signal on neutrophils and may be released by astrocytes and activated microglia both to promote microglial migration and to restrain VNR-mediated microglial phagocytosis (Cardona et al., 2006; Noda et al., 2011; Jeon et al., 2012). The protein C-X3-C motif ligand 1 (CX3CL1; also known as fractalkine) is usually localized on the neuronal cell surface, where it may participate in suppressing microglial inflammatory responses by activating the microglial CX3CR1. Yet, nerve injury or excitotoxicity results in the cleavage of membrane-bound CX3CL1 and the release of its soluble form, which attracts microglia and may stimulate microglial phagocytosis of PS-exposed neurons by increasing the release of the bridging protein, MFG-E8 (Cardona et al., 2006; Noda et al., 2011).

### Cytokine Release

Microglia produce and secrete low molecular weight proteins, the cytokines, that include ILs, interferons, tumor necrosis factors (TNFs), CSFs and TGFs. Cytokines are involved in cellular communication, regulate inflammation and immune responses, and cell growth, survival and differentiation. In microglia the expression and secretion of cytokines is well documented both *in vitro* and *in vivo* and the events that promote their production are numerous and include bacterial cell wall components, viral envelopes, prion protein, proteoglycans and (lipo)teichoic acid, growth factors or cytokines such as macrophage-colony stimulating factor (M-CSF) and granulocyte-macrophage (GM)-CSF, inflammatory mediators such as the plated-activating factor and complement components, high ATP levels as well as high extracellular potassium concentrations, serum proteins, amyloid β (Aβ) protein and DAMPs (Nakajima and Kohsaka, 2001; Hanisch, 2002).

Under non-stimulated conditions, human microglia express numerous cytokines including IL-1β, IL-6, IL-8, IL-10, IL-12, IL-15, TNF-α, macrophage inflammatory protein (MIP)-1α, MIP-1β and MCP-1; in addition, they express cytokine receptors like IL1-RI, IL1-RII, IL-5R, IL-6R, IL-8R, IOL-9R, IL-10R, IL-12R, IL-13R IL-15R, TNFRI, TNFRII and gp130, while the expression of IL-11R, IL-4R, IL-2R, IL-3R, IL-7R have never been reported (Kim and de Vellis, 2005).

After LPS stimulation the expression of all cytokines is dramatically increased, except for IL-15 (Kim and de Vellis, 2005). IL-1β is a crucial cytokine in immune stimulatory and pro-inflammatory signaling and plays a role in innate defense and immune response; the targets of IL-1 are T cells, B cells, monocytes, macrophage and microglia themselves. In the CNS, activated microglia represent the principal source of IL-1 during infection, ischemia, stroke, and excitatory and mechanic injury. Furthermore, in addition to its role in inflammation, IL-1 influences cell proliferation and differentiation during CNS development and can modulate synaptic efficacy in neuronal populations, in particular in the hippocampus (Giulian et al., 1988; Vitkovic et al., 2000) (**Table 1**).

**Table 1:** Cytokines produced and released by activated microglial cells.

Cytokines	Functions	Reference
IL-1	Involvement in innate defense and immune response; cell proliferation and differentiation during CSN development; modulation of synaptic efficacy (hippocampus)	Giulian et al., 1988;Vitkovic et al., 2000
IL-15	Cell survival	Hanisch et al., 1997
IL-10	Modulation of IL-1β and TNF-α production and release; modulation of cytokine receptor expression	Sawada et al., 1999
IL-6	Fever induction; neuroendocrine mobilization of energy stores; reduction of food intake; increase of pain perception and sleep; activation of astrocytes	Raivich et al., 1999
TNF- α	Promotion of inflammation and edema; induction of glutamate release from astrocytes; neuron survival (at low concentrations)	Carlson et al., 1999;Bezzi et al., 2001
TGF-β	Reduction of pro-inflammatory cytokine production; wound healing; reduction of AD plaque formation in animal model	Wyss-Coray et al., 2001
IFN-γ	Upregulation of several cell surface molecules (MHC-I, MHC-II); modulation of proteasome composition, cytokines, complement proteins and NO release-induced apoptosis	Badie et al., 2000;Hanisch, 2001

IL-15 can replace some functions proper of IL-12 and it is known that microglia produce IL-15; moreover microglial cells also express IL-15 receptor and, *in vitro*, the stimulation of microglial cells with IL-15 causes an increased cell survival (Hanisch et al., 1997) (**Table 1**). Contrariwise, IL-10, IL-4 and TGF-β act as anti-inflammatory, immunosuppressive and neuroprotective cytokines, mediating the downregulation of IL-1β and TNF-α expression or attenuating their effects; these cytokines also modify the expression of microglial cell surface molecules (Sawada et al., 1999) (**Table 1**). Besides reducing pro-inflammatory cytokine and chemokine production, TGF-β plays a role in tissue development and wound healing. TGF-β1 was shown to reduce AD plaque formation in an animal model of AD (Wyss-Coray et al., 2001) (**Table 1**).

IL-6 is a pro-inflammatory cytokine that initiates and coordinates the inflammatory responses, thus avoiding the spread of infectious agents; furthermore, in CNS, in addition to fever induction, IL-6 plays a role in neuroendocrine mobilization of energy stores, reduction of food intake, increase of pain perception and sleep. It should be highlighted that IL-6 can have both pro-inflammatory and anti-inflammatory outcomes and these effects depend on the simultaneous presence of other cytokines; in fact, microglial cells release IL-6 in early phases of CNS injury, and thereafter IL-6 acts on astrocytes to activate them to repair damaged tissue (Raivich et al., 1999) (**Table 1**).

TNF-α is another pro-inflammatory cytokine released by neurons, astrocytes and microglial cells. Ischemia, injury, bacterial and viral infections, multiple sclerosis and AD increase the expression of TNF-α in brain; in these conditions microglial cells represent the most prominent source of TNF-α. TNF-α promotes inflammation and edema and can induce astrocytes to release glutamate (Bezzi et al., 2001). In addition, at high concentrations TNF-α is toxic to neurons and myelin, but at low levels it promotes neuronal survival (Carlson et al., 1999) (**Table 1**).

Type I interferons (IFNα, IFNβ, INFω and IFNτ) are expressed in the CNS after infection, while high levels of IFNγ are detected in other pathological conditions. IFNγ upregulates LPS receptor, intercellular adhesion molecule-I, MHC class I and II, leukocyte function-associated molecule 1, immune-accessory molecules B7 (CD80/86), (CD14), Fc and complement receptors. Furthermore, IFNγ acts to change the proteasome composition and the release of cytokines (IL-1β, TNF-α and IL-6), complement proteins (C1q, C2, C3 and C4) and NO and to induce apoptosis through Fas and FasL upregulation (Badie et al., 2000; Hanisch, 2001) (**Table 1**).

## Role of Microglia in Neuronal Development Immunosurveillance

Microglial cells of the healthy CNS have been considered “resting” for decades, with the purpose to distinguish them from the activated status in injured or diseased CNS. Currently, the improvements in the two photon imaging technique have allowed to demonstrate that, in transgenic mice that overexpress enhanced GFP in the *Cx3cr1* locus, microglial cells repeatedly examine the environment and that their processes and arborization are highly mobile. Furthermore, it was demonstrated through time-lapse imaging, that while the somata of microglial cells remain in the same position, the microglial processes undergo cycles of *de novo* formation and withdrawal and directly contact astrocytes, neurons and blood vessels to monitor minimum changes in their microenvironment. The dynamic reorganization of microglial processes may be considered a housekeeping function by which microglial cells monitor the environment, remove metabolic products and deteriorated tissue components, and recognize neuronal activity and structural alterations, in order to preserve and organize neuronal networks (Nimmerjahn et al., 2005). Thus, the term “surveillant” has been proposed to describe how microglial cells actively and continuously monitor the healthy CNS (Ransohoff and Cardona, 2010). Surveillant microglial cells constitutively express low levels of Human Leukocyte Antigen-D Related in human CNS and MHC-II in rodent CNS, which points to the ability of these cells to present antigens and confirms their central role in immune surveillance. Moreover, even minor pathological events induce an increased expression of MHC-II, CD89, CD86, CD40, CD11a, CD54 and CD58, considered molecular markers of antigen presentation and activation, thus confirming that microglial cells are able to present antigen and to activate T cells. In the healthy CNS microglial cells are retained in a surveillance state through the interaction between the CD200 and CD200 receptor (CD200R), CD22-CD45 (also known as PTPRC), CD172A (also known as SIRP-α)-CD47 and CX3CL1-CX3CR1. CD22-CD45 and CD200-CD200R signals require cell–cell contact, whereas the CX3CL1-CX3CR1 signal can occur by cell–cell contact or at distance through the release of soluble CX3CL1 (Ransohoff and Cardona, 2010). The inhibitory CD200-CD200R signaling was demonstrated in CD200-deficient mice, in which microglial cells show a less ramified morphology and enhanced CD11b and Cd45 expression (Hoek et al., 2000). Neuronal electrical activity also acts to suppress MHC-II expression on surrounding microglial cells and astrocytes (Neumann, 2001).

In addition to their role in antigen presentation, microglial cells are able to recognize factors that in physiological conditions are not present in the parenchyma (microbial structures and serum components) and of factors that are present above a threshold concentration (intracellular components) or are present with an abnormal structure (protein aggregates). However, it is important to underline that the transition from the surveillant to the activated status of microglial cells represents a change in functional phenotype rather than an awakening; indeed, chemotactic reorientation and non-transcriptional modification take place within minutes and then in few hours a massive induction of gene expression occurs.

Activation of microglial cells results in different phenotypic changes (enlarged soma, retracted and shortened processes and increased expression of myeloid markers) that reflect functional diversity; indeed, when stimulated by microbial components microglial cells react by activating a phagocytosis program and releasing pro-inflammatory cytokines, whereas when these cells are challenged by apoptotic cells or myelin debris they respond by releasing anti-inflammatory mediators (Hanisch and Kettenmann, 2007). Another feature of activated microglial cells, *in vivo*, is their proliferative activity. This characteristic has been studied *in vitro* and the factors responsible of mitogenesis have been identified; specifically, *in vivo*, microglial cells proliferate under the stimulus of GM-CSF, M-CSF and IL-3 while, *in vitro*, IL-2, IL-4, IL-5 and TNF-α can induce microglial proliferation (Nakajima and Kohsaka, 2001).

## Non-Conventional Roles

del Rio-Hortega (1932) considered amoeboid, ramified and reactive microglia as different forms of a single cell type. Amoeboid microglia were considered as active macrophages during development and precursors of resting or ramified cells. Resting or ramified microglia could, in response to infection, traumatic injury, or ischemia, reactivate in the postnatal brain, assume an amoeboid morphology, and migrate to the site of injury. In this context, ramified microglia were thought to contribute to the well-being of neurons, without other specific functions, while amoeboid microglia were thought having a passive scavenger function by removing the cells that die during the course of normal development and remodeling of the fetal brain. CNS insults such as microbial invasion induce dramatic morphological changes, from ramified to amoeboid microglia, with a quick up-regulation of different receptor types and production of many secretory factors that contributes to the defense of the brain. Since a long time, this point of view has changed.

During the last years a great interest about the role of microglial cells in normal brain development has emerged and many studies have explored how these cells participate to model the developing CNS. Microglial cells participate in the control of programmed cell death (PCD) that occurs during the early postnatal development; the involvement of these cells in PCD was supposed following the observation that, in vertebrates, half of neurons are eliminated during development and that, in developing retina and hippocampus, microglial cells are present in zones of neuronal expansion, often close to apoptotic neurons (Ashwell et al., 1989; Oppenheim, 1991). Microglia act phagocytizing dead or dying neurons and cellular debris. One of the first report documenting the phagocytic role of microglial cells, which used the silver impregnation method of del Rio-Hortega, evidenced the presence of amoeboid phagocytic cells in the II/III cortical layer and subplate during the first postnatal week after birth that is the period of time during which high levels of PCD were reported in these regions (Ferrer et al., 1990). While our knowledge is becoming larger, the mechanisms by which microglial cells phagocytize dead or dying neurons are still not completely understood (Miyamoto et al., 2016).

In addition to the involvement of microglial cells in the removal of dying neurons, these cells can also participate in the tissue remodeling process and cell death. The first evidence of this role derives from the peripheral nervous system, where macrophages have been proven to be necessary for the elimination of transient structures in the mouse eye. More in detail, after ablation of macrophages a population of ocular cells which usually disappears during a precise time line, persists for several days (Lang and Bishop, 1993).

Currently, a growing body of evidence about the role of microglial cells in PCD induction in the CNS has definitely emerged. In fact, in embryonic explants of the rat spinal cord, microglial cells appear to be necessary for motor neuron PCD as they release TNF-α, that, by acting on receptor 1 expressed on motor neurons, induces their death (Sedel et al., 2004). On the other hand, microglial cells are also involved in Purkinje neurons’ PCD and in the degradation of internalized pieces of neurons which occur through the production of superoxide ions during respiratory bursts (Marín-Teva et al., 2004). These *in vitro* data have been confirmed by *in vivo* studies performed using transgenic mice deficient for CD11b, integrin and DAP12 immunoreceptor. These mice display decreased apoptosis in hippocampus, which further corroborates the hypothesis that the proper functioning of microglial cells is crucial for PCD (Wakselman et al., 2008). In addition, a recent study in transgenic mouse has shown that in the developing cerebral cortex neuron–microglia interaction through the Cxcl2/CxcR4-7 axis is fundamental for the proper microglia (Arnò et al., 2014).

In rat, cerebellum reaches maturation during the first 3 postnatal (P) weeks and undergoes remarkable anatomical changes during this time (Sillitoe and Joyner, 2007; Butts et al., 2014). Microglia dramatically change their morphological profile in the cerebellar cortex during this dynamic period of development. In this context a continuous process of microglial maturation occurs and this might be related to specific functions including regulation of cell death and synaptic pruning. Microglia morphology change from amoeboid to ramified in a region specific manner with a sudden appearance of phagocytic cups at P days 17–19 (Perez-Pouchoulen et al., 2015). Previous evidence of microglial phagocytosis in the developing cerebellum focused on amoeboid (activated) microglia which would play a non-specific role to improve the structural plasticity of the medulla during cortical folding, and/or to wipe the path over the course of cortex development, (Ashwell, 1990). Conversely, ramified (quiescent) microglia are the dominant committers of phagocytosis during the second and third postnatal week of development in the cerebellum, which suggest a specific role (Perez-Pouchoulen et al., 2015). However, how phagocytic microglia contribute to the establishment of the cerebellar circuit remains poorly understood.

Conversely, microglial cells are able to promote the proliferation of neural precursor cells (NPCs) and neuronal survival. In this regard, the conditioned medium of cultured microglial cells was shown to promote NPC proliferation, neuronal survival and maturation (Nagata et al., 1993). Besides, a recent *in vitro* study has shown that cultures of NPCs derived from mice lacking microglial (*Pu^-/-^*) cells displayed decreased proliferation, but when microglial cells from wild type mice were added to the culture, cell proliferation was restored (Antony et al., 2011). The involvement of microglial cells in neuronal survival was also demonstrated in *in vivo* studies. Indeed, when mouse microglial cells are inactivated, it is possible to observe an increased neuronal apoptosis in layer V of the cerebral cortex between P3 and P5. This suggests that during this stage of development, microglial cells play a pivotal role, providing trophic support, at least in part through IGF-1 signaling, which is a downstream target of the fractalkine receptor (CX3CR1) (Ueno et al., 2013).

Along with the role of microglial cells in reducing the excess of neurons occurring at the beginning of CNS development, microglial cells are also crucial in the process of synaptic pruning, function and maturation (Kano and Hashimoto, 2009). In order to analyze microglia interactions with neuronal synapse, Hirasawa and colleagues created transgenic mice expressing EGFP in microglial cells and neurons (Iba-1-EGFP/Thy1-EGFP M line) (Hirasawa et al., 2005). They observed that in layers II/III of somatosensory and visual cortex of young adult mice, microglial cells directly contact synapses with a median frequency of about one per hour and that a reduction of neuronal activity results in a decreased frequency of microglia-synapse contact (Wake et al., 2009). Another study combining two photon microscopy with 3D section electron microscopy reconstruction techniques, confirmed that in layer II of visual cortex, microglial cells contact spines, presynaptic terminals and synaptic clefts; in particular, microglial cells contact smaller spines and these spines are those that disappear in later images (Tremblay et al., 2010).

Several works have demonstrated that microglia engulf and eliminate synapses during development (Paolicelli et al., 2011). In developing brain of mice lacking CX3CR1, synaptic pruning is delayed probably due to reduction of microglial cell number. These mice show a high spine density in hippocampus during postnatal weeks 2 and 3 and an increased immunoreactivity for the postsynaptic density protein 95 (PSD95), revealing that the synaptic pruning is impaired with immature synapses characterized by peculiar pharmacological and electrophysiological features of immature brain. Fractalkine turns out to be essential for the activity of microglia. On the one hand it could act by enhancing microglia migration and proliferation, at least during development; in this case the number of microglial cells in the brain would be reduced and the capacity of synaptic pruning limited, in mice lacking CX3CR1. On the other hand, fractalkine could be essential for the identification of synapses to be removed by the microglia; in this case the efficacy of synapse removal would be reduced (Paolicelli et al., 2011). It is common ground that other signals could be involved in this process, such as component cascade complement C1q and C3.

Moreover, in the mouse retinogeniculate system, during the first weeks of postnatal development overlapping inputs are removed and eye-specific territories are formed; indeed, if neuronal activity is damaged or one eye is removed this pruning mechanism is impaired (Chen and Regehr, 2000; Huberman et al., 2008). To further analyze, *in vivo*, the involvement of microglial cells in synaptic refinement and eye specific segregation, the cholera toxin, conjugated with red or blue fluorescent dye to distinguish left from right eye inputs, was administered in transgenic mice (*Cx3cr1^GFP^)* to label and identify retinal ganglion cell (RGC) inputs originating from each eye. In this assay microglial cells were observed to engulf presynaptic RGC inputs during the peak of pruning, this process decreasing after P10, when eye specific territories are well formed. Furthermore, when activity-dependent synaptic competition was induced through the administration of tetradotoxin or forskolin in order to silence or boost activity, the less functional eye loses territory in dorsal lateral geniculate nucleus (dLGN) and the axon terminals were engulfed by microglial cells (Schafer et al., 2012).

Taken together, these data confirm that microglial cells are key mediators in the process of synaptic pruning but the molecular mechanisms by which these cells tag synapses for elimination are still not completely known. The classical complement cascade has been supposed to be involved in microglia-synapse interaction because in the retinogeniculate system the complement proteins C1q and C3 are localized to synaptic compartments and participate in synaptic pruning (Stevens et al., 2007). Consistently, it was demonstrated that in the postnatal dLGN, C3 protein is expressed and localized to a subset of synapses, while C3 receptor (CD11b) is expressed at high levels by microglial cells at P5. Additionally, C3 or C3R knockout mice show deficit in eye specific segregation because there is a reduced engulfment of retinal axons, confirming that microglial cell phagocytosis is crucial for synapse elimination (Schafer et al., 2012). Moreover, C1q and C3 specifically bind less-active synapses for removal by microglia via CR3 (Stevens et al., 2007). How synapse activity regulates complement proteins remains unknown. Notably, in the hippocampus, during hypoxic injury and inflammation, microglial CR3 was required to induce long-term synaptic depression (Zhang et al., 2014). This suggests that microglia regulate synaptic plasticity via CR3 not only in physiological condition, but also in altered and harmful environments.

Microglia have also been involved in the early wiring of the embryonic brain. Recent work in the embryonic mouse demonstrated that microglia appear to engulf a subset of dopaminergic axons (Squarzoni et al., 2014) and the number of these axons was increased in mice lacked microglia. Conversely, LPS-activated microglia caused a decrease in dopaminergic axons (Squarzoni, et al., 2014). In another study, the consequences of microglial dysfunction on the formation of corpus callosum were evaluated. Using *Dap12^-/-^* mice and a maternal inflammation model by administering LPS to E15.5, alterations of callosal axons were highlighted, demonstrating the importance of microglia on the development of this structure (Pont-Lezica et al., 2014).

The development of other CNS cell types is also affected by microglial functions. Regarding glial cells, culture medium conditioned by microglia promotes both the survival and the differentiation of oligodendrocyte precursors into mature oligodendrocytes through IGF-1, NF-κB, IL-1β, and IL-6 (Shigemoto-Mogami et al., 2014). Moreover, *in vitro* experiments suggest that neural stem/precursor cells may be pushed to differentiate into astrocytes by microglia-conditioned media by means of IL-6 and leukemia inhibitory factor (Nakanishi et al., 2007). Microglia have also been suggested to promote myelination (Clemente et al., 2013). These outcomes suggest that microglia could potentially regulate maturation, survival and proliferation of most of developing CNS cell types.

Increasing evidences show that microglia positively regulate the vascularization of developing retina (Santos et al., 2008) and consistently, *in vitro* and *in vivo* depletion of microglia suggests that microglia are required for retina and hindbrain vascular branching (Checchin et al., 2006). However, other works have described the opposite effect (Unoki et al., 2010). Thus, additional information is required to precisely determine how microglia regulate vasculogenesis and whether this action is limited to the developing retina or is a more general effect occurring in the CNS.

## Role of Microglia in Neurodevelopmental Diseases

**FIGURE 4:**
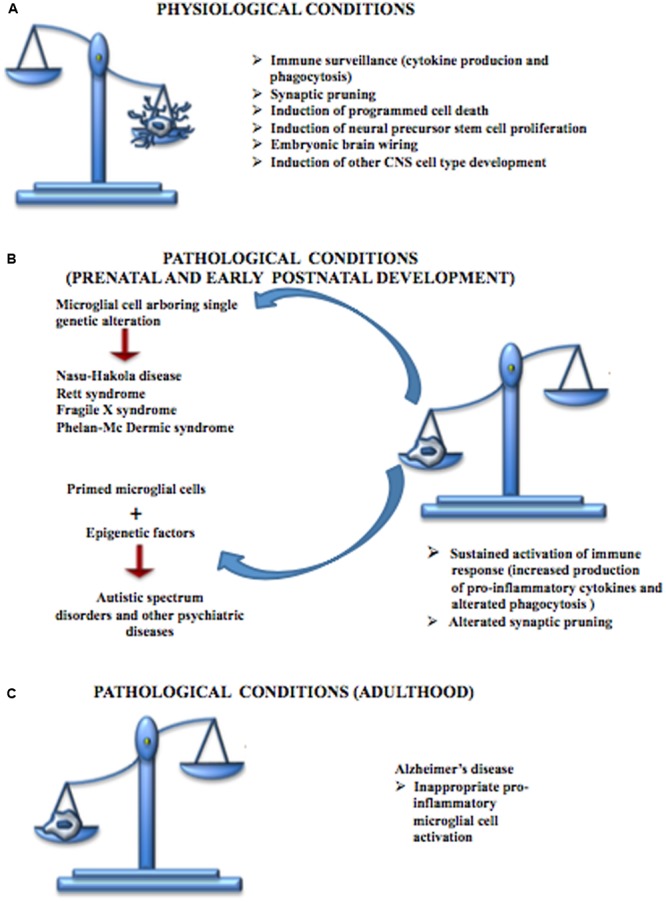
Contribution of microglial cells in physiologic and pathologic conditions. **(A)** During CNS development, microglial cells are responsible for the immune surveillance and are involved in the regulation of the development of other CNS cell types, neural stem cell proliferation, neuronal programmed cell death, embryonic brain wiring and synaptic pruning. **(B)** Microglial cells play a central role in many neurodevelopmental diseases. Some of these disturbances, such as Nasu-Hakola disease, Rett syndrome, Fragile X syndrome and Phelan-Mc Dermic syndrome are characterized each by one well known genetic alteration while others such as Autistic Spectrum Disorders originate from the interactions between genetic alteration arbored by microglial cells and epigenetic factors; however, both conditions are characterized by a sustained production of pro-inflammatory cytokines, alterations in phagocytosis mechanisms and defective synaptic pruning. **(C)** Microglial cells are also involved in some CNS diseases typical of the adult such as Alzheimer’s disease, characterized by inappropriate pro-inflammatory immune response.

Neurodevelopmental diseases arise early in life and are often characterized by intellectual disability, social deficit, and obsessive–compulsive and other disorders including motor abnormalities. The pathophysiology of these disorders is unclear, although altered synaptic transmission within the cortex is a common event. Therefore, deficits in synaptic maturation characterized by weak functional connectivity or an excess of weak excitatory synapses may have a role in those mental diseases. Microglia have a critical role in pruning synapses during development. Altered function of these cells has been proposed to participate in the pathogenesis of these disorders (**Figure 4**). Recently the term “microgliopathy” has been suggested, acknowledging that dysfunction of microglia can represent a primary disease-causing mechanism (Prinz and Priller, 2014).

**FIGURE 5:**
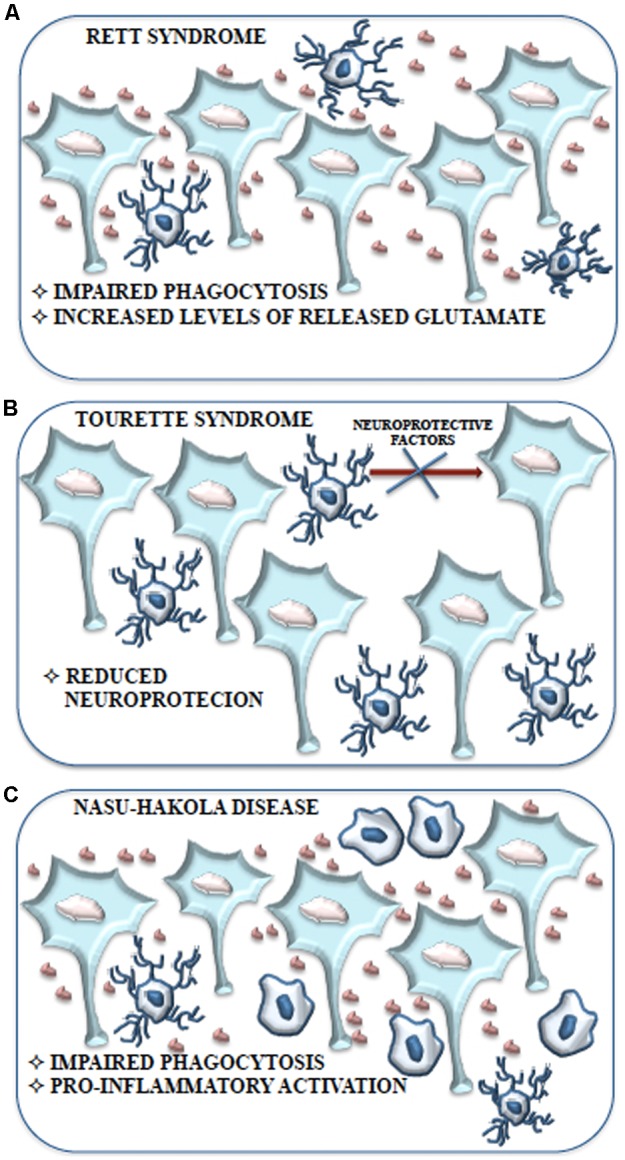
Schematic representation of the contribution of microglial cells to Rett syndrome, Tourette syndrome and Nasu-Hakola disease. **(A)** Impaired phagocytosis due to alteration or loss of *Mecp2* expression and the consequent accumulation of neuronal debris contribute to the development of Rett syndrome without the induction of a pro-inflammatory response. Moreover, the high levels of glutamate produced and released by microglial cells may cause the adverse effects on neuronal function observed in Rett syndrome. **(B)** The behavioral and neurochemical abnormalities observed in Tourette syndrome might be associated with a reduction of microglial cells expressing IGF-1 that results in an impaired neurotrophic protection of neurons. **(C)** Aberrant expression of TREM2 and DAP12 in microglial cells is considered tightly related to the inhibition of apoptotic neuron clearance observed in Nasu-Hakola disease. Impaired phagocytosis could be responsible for the excessive pro-inflammatory activation of microglial cells resulting in neurodegeneration with amyloid plaque deposition and early FTD.

NHD, or polycystic lipomembranous osteodysplasia with sclerosing leukoencephalopathy, is characterized by four stages: latent, osseous, early neurologic, and late neurologic (Kaneko et al., 2010). NHD is a rare intractable autosomal recessive leukodystrophy (Hakola, 1972) caused by genetic mutations of either DAP12 or TREM2 (Paloneva et al., 2002) and is considered as a type of “primary microgliopathy.” During the initial neurologic phase, usually in the third or fourth decade of life, patients develop personality changes and mild memory impairment. In the late neurologic phase, patients progress to dementia that generally leads to death in the fifth decade of life. DAP12 and TREM2 form a signaling complex expressed exclusively in microglia, macrophages, dendritic cells and osteoclasts. The DAP12 pathway is characterized by phosphorylation of the downstream effector Syk. Recent works have shown an increase in Syk phosphorylation in microglia and neurons of NHD patients compared to healthy control (Satoh et al., 2012). These data point to microglia activation likely due to unrestricted Syk activation in the absence of functional TREM2 and DAP12 complex. In addition, TREM2 is strongly expressed in AD brain tissue, notably close to amyloid plaques and can mediate amyloid plaque phagocytosis. When in AD patients TREM2 and/or DAP12 are not functional, amyloid engulfment fails determining a similar amyloid associated AD-type dementia to that observed in NHD (Xiang et al., 2016). These conditions would cause accumulation of amyloid-β plaques with secondary and undesirable pro-inflammatory microglial activation. Brain CT and MRI reveal prominent activation of microglia in the temporal and frontal white matter in NHD patients (Nwawka et al., 2014). In unstimulated microglia, TREM2 is highly expressed and its expression strictly overlaps with DAP12 gene expression (Paloneva et al., 2002) and is downregulated by LPS and IFN-γ (Schmid et al., 2002). TREM2 expression in microglia promotes phagocytosis of apoptotic neurons producing very small quantities of pro-inflammatory cytokines (**Figure 5**). However, only microglial clusters and neuron-associated microglia (Lue et al., 2015) express TREM2. Apoptotic neurons induce undue pro-inflammatory responses and microglial activation leading to neuroinflammation, if TREM2 is not expressed. Thus, defects in DAP12 or TREM2 function in NHD microglia inhibit clearance of apoptotic neurons and are believed to play a central role in the NHD pathogenesis (**Figure 5**). In this case, TREM2 or DAP12 deficiency may lead to excessive pro-inflammatory microglial activation that causes neurodegeneration with amyloid plaque deposition. Given the strong evidence supporting the role of TREM2 and DAP12 inhibitory TLR signals (Ito and Hamerman, 2012), it is possible to hypothesize that in NHD, amyloid-stimulated TLR activation is disinhibited, with consequent excessive microglial activation and reduced ability to phagocytose amyloid deposits or apoptotic neurons.

Hereditary diffuse leukoencephalopathy with spheroids (HDLS) is an autosomal dominant disease and can be considered a microgliopathy with similar features to NHD. Disease onset occurs around a mean age of 40 years with a course that leads to death within 6 years. *CSF1R* defects have been highlighted in HDLS as a cause of the disease (Rademakers et al., 2011; Stabile et al., 2016). In the brain, under physiological conditions, CSF1R is expressed exclusively in microglia and has two ligands, CSF1 and IL-34. The CSF1/CSF1R interaction leads to the formation of homodimer receptors with subsequent autophosphorylation. This determines the CSRF1’s full activation and phosphorylation of different kinases, including Src, ERK, Akt. The CSF1/CSF1R axis regulates the proliferation, differentiation and survival of microglia. Indeed, *Csf1r* knockout mice are devoid of microglia and die before the adulthood. Contrariwise, in the adult the use of CSF1R specific inhibitors causes the depletion of microglia, but this neither has adverse effects nor causes behavior disorders (Elmore et al., 2014). In HDLS the alteration of the CSF1/CSF1R axis could lead to the blockade of CSF1R kinase activity resulting in alterations of phosphorylation of downstream targets.

A recent work (Otero et al., 2009) showed that CSF1R leads to stabilization and nuclear translocation of β-catenin resulting in increased proliferation and cell survival. In this case DAP12 is crucial. Indeed, *Dap12* deficient mice show a decreased presence of microglia in specific areas of the CNS, and a lower stabilization and nuclear translocation of β-catenin, probably due to a decreased CSF1R activity. Therefore, DAP12 modulates CSF1R activity, most likely through TREM2. The DAP12/TREM2/CSF1R cross-talk could explain some common traits between NHD and HDLS.

Recently Rett syndrome (RTT) microglia have been proposed to have a pathological role albeit outside the context of immune activation. RTT is one of “syndromic” autistic spectrum disorders (ASDs), a group of genetically different neurodevelopmental disorders with high penetrance of ASD diagnosis, characterized by clear metabolic and genetic anomalies that are eligible to animal modeling (Levitt and Campbell, 2009). RTT is caused by loss of function of the X-linked *MECP2* gene. MeCp2 is an epigenetic modulator with the ability to bind CpG dinucleotide in target genes to modulate chromatin structure and gene transcription (Chahrour and Zoghbi, 2007). RTT affects young girls between 8 and 16 months of age and is characterized by decelerated brain growth accompanied by loss of motor abilities, ataxia, loss of cognitive capability, respiratory dysfunction and autistic features; this regressive course is comparable to that observed in autism and both diseases show dendritic and synaptic abnormalities (**Figure 5**), suggesting that MeCp2 aberration might be involved also in autism (Shepherd and Katz, 2011).

Contrary to a common belief, RTT is not only due to defect in *MECP2* in neurons. Up-to-date results demonstrated glial involvement in the pathology. In particular, in normal physiological conditions neurons and other cells, including microglia, show high MeCp2 expression (Maezawa and Jin, 2010). It was demonstrated that *Mecp2^-/-^* microglia present a striking impairment of phagocytic capability as compared to controls (Derecki et al., 2012) and analysis of *Mecp2^-/-^* brain tissue shows increased levels of cellular debris. The poor removal of debris could support the onset and maintenance of Rett pathology (Derecki et al., 2012) (**Figure 5**). Moreover, conditioned medium of mixed glial cultures, obtained from *Mecp2^-/-^* mice, shares a toxic activity and this activity is lost in *Mecp2^-/-^* cell cultures in which microglial cells are eliminated. Indeed, pure *Mecp2^-/-^* microglial cultures release high level of glutamate in the culture medium and it is known that, at high concentration, glutamate is able to cause stunted dendritic morphology and synaptic loss (Maezawa and Jin, 2010) (**Figure 5**). However, this condition would not be accompanied by an increase in pro-inflammatory cytokines, NO and prostaglandin E2 that characterize the induction of an inflammatory process, suggesting that microglia are able to induce neurotoxicity due to intrinsic dysfunction. These findings are in accordance with the absence of microgliosis in RTT brains and with the observation of increased glutamate levels in both brain and cerebrospinal fluid (Hamberger et al., 1992; Jellinger, 2003; Ballas et al., 2009). High levels of glutaminase have been demonstrated in *Mecp2^-/-^* microglia (O’Driscoll et al., 2013). Glutaminase causes dendritic and synaptic damage and this has been proposed to be an additional and important aspect in RTT (Maezawa and Jin, 2010). The upregulation of glutaminase may occur through MeCp2-dependent NF-κB activation (Mann et al., 2007). Thus, in RTT, a dysregulated inflammatory response driven by the MeCp2-NF-κB axis and an unrestrained glutamate production (i.e., high glutaminase expression) could contribute to and exacerbate the disease (O’Driscoll et al., 2013) (**Figure 5**). Besides, *Mecp2^-/-^* mice that received bone marrow transplant displayed remarkably blunted pathology and a robust engraftment of microglia-like cells in brain parenchyma was detected. Improved activity of microglia was therefore associated with blunted pathology. Complementary genetic/pharmacological approach using *Mecp2*^lox-stop/y^*Lysm*^cre^ mice (Derecki et al., 2012) confirmed these results. Blocking debris uptake by administration of annexin abolished the arrest of the disease, strongly suggesting that the disease had been halted by the ability of bone marrow transplant to generate new functional microglia with ability to remove debris (Derecki et al., 2012).

The results of Derecki et al. (2012) were confirmed by Cronk et al. (2015) which have shown as in *Mecp2^-/-^* mice transcriptional alterations and loss of macrophages in different tissues are evident. Thus, MeCp2 could be involved in a more general control mechanism of the inflammatory response in cells belonging to the macrophage/monocyte lineage, including microglia (Cronk et al., 2015), justifying the complexity of the symptoms associated with RTT.

Unfortunately, Wang et al. (2015) have not confirmed these results, which gave rise to an ongoing controversy. In an attempt to reproduce the results of Derecki et al. (2012), Wang et al. (2015) did not observe any benefit to *Mecp2^-/-^* mice following wild-type bone marrow transplant and could not replicate the results reported by Derecki et al. (2012). The question remains whether and how microglia participate in the pathogenesis of RTT. A recent work contributed to elucidated this aspect in part (Schafer et al., 2016). Using the retinogeniculate system of *Mecp2^-/-^* mice and Cre-lox technologies to express or ablate *Mecp2*, these authors showed that during late stages of the disease microglia participate in the destruction of the neuronal circuitry already made vulnerable by the lack of expression of MeCp2 in neurons. These authors concluded that the discrepancies between the Derecki et al.’s (2012) and Wang et al.’s (2015) results could be partly explained by the different levels of myeloid-derived cell-specific MeCp2 expression.

However, the *LysM^Cre^* model used by Derecki and Wang was not really specific to microglia due to a recombination in all kind of cells of the myeloid lineage and only 30–40% in microglia. Moreover, being adult microglia considered LysM-negative the recombination event probably occurs during development. The conditional tamoxifen-inducible *Cx3cr1^CreER^* model used by Schafer and Cronk is more specific to microglia than the *LysM^Cre^* line (higher than 90% recombination) and avoids the unknown effects of peripheral myeloid cells. This model has effectively improved microglia targeting in RTT.

Tourette syndrome (TS) is a neurodevelopmental tic disorder viewed as a spectrum, encompassing other stereotyped behaviors with socially inappropriate remarks or gestures. TS is associated with developmental abnormalities of cortico-subcortical and intracortical networks that are responsible for the selection and inhibition of motor output and sensory input. Its etiology is complex, with potential involvement of both environmental factors and polygenic contribution. Recent work has identified a mutation in *L-histidine decarboxylase (HDC)*, which encodes the rate-limiting enzyme HDC in the biosynthesis of histamine, as a rare but high-penetrance genetic cause of TS (Ercan-Sencicek et al., 2010). Knockout of the *Hdc* gene, which recapitulates this molecular abnormality, thus produces an animal model with strong etiologic validity. These mice exhibit behavioral and neurochemical abnormalities seen in patients with TS, further confirming the validity of the model (Castellan Baldan et al., 2014). In this model abnormalities in microglia activation, such as reduced arborization have been recently described (Frick et al., 2016). The total number of microglia is unchanged in KO animals, but the number of microglia expressing IGF-1 is reduced and this suggests impaired neurotrophic protection of neurons (**Figure 5**). Indeed, reduced IGF-1-expressing microglia have been found in the striatum of *Hdc^-/-^*mice and of TS (Frick et al., 2016). IGF-1^+^ microglial cells are required for neuronal support during postnatal development, at least in the cortex (Ueno et al., 2013; Ziv et al., 2016). Inflammatory challenge with LPS dramatically changed this pattern: microglia activation in the striatum was enhanced in *Hdc^-/-^* mice, compared with wild-type controls. This was accompanied by enhanced induction of the pro-inflammatory cytokines IL-1β and TNF-α. Taken together, these findings suggest that in this model there is a deficit in microglia-mediated neuroprotection, accompanied by over-reactivity to environmental challenge (Frick et al., 2016). After LPS challenge, microglia activation in the animal model much more closely resembles that seen postmortem in humans.

Microglia have a key role in synaptic pruning during development, with long lasting consequences in adulthood (Paolicelli et al., 2011; Schafer et al., 2012). In the *Hdc^-/-^* mouse, striatal microglia present morphological abnormalities (Frick et al., 2016). Possibly, microglial impairments in this animal model lead to alterations of synaptic pruning. Besides, *Hdc^-/-^* animals may have CX3CR1 deficiencies, which could be associated with abnormal synaptic pruning (Zhan et al., 2014).

Regarding the long-term effects, animal models suggest that premature and inappropriate activation of the immune system, e.g., neonatal exposure to bacterial infections, could adversely affect the physiological development of the CNS, even immediately after exposure (Bilbo and Schwarz, 2012). In this case, long-term consequences on stress responses, the proper immune response that develops, and behavior altered social interactions could occur (Spencer et al., 2006; Bilbo, 2010). However, these results need to be confirmed in TS patients even if preliminary evidence from post-mortem and animal model studies shows that microglia could have a direct role in TS and other behavioral patterns.

Autistic spectrum disorders (ASDS) include many developmental disabilities defined by repetitive behaviors, communication difficulties and deficits in social interactions. The neurobiological basis of these disturbances is still not exactly understood but seems to be a complex combination of genetic alteration, epigenetic regulation, environmental factors, glial cell abnormalities, aberrant neurogenesis and auto-antibodies production. To date, due to the lack of knowledge about the pathogenesis of these disorders there is no cure for ADSs, but only symptomatic treatment. Recent work demonstrates the existence in ASD of a relationship between neural development and immune cells (Depino, 2013; Frick et al., 2013). Understanding this relationship, from prenatal to postnatal periods, could have clinical relevance.

There are hundreds of genetic variants involved in the causation of idiopathic autism. Monogenic diseases include Fragile X syndrome, *SHANK3* gene deficiency (Phelan-Mc Dermid syndrome), RTT and others. The genes that have been linked to ASDs are commonly related to synaptic and immune functions (Voineagu et al., 2011) and induce common pathological processes such as inflammation, abnormal microglial functions, and synaptic dysfunction. Strong evidences have recently related alterations of neural synaptogenesis and immune pathways to ASD phenotype, but to date the link between the possibility to develop autism and these processes is unclear. However, many evidences have suggested a relationship between inflammatory processes and consequent destruction of neuronal circuitry (Mead and Ashwood, 2015). Inherent abnormalities in microglia may link these two processes, i.e., inflammation and altered neural networks.

The data currently available about the involvement of microglia in autism are often limited to the morphological analysis of these cells in a small number of patients. Vargas et al. (2005) first described in postmortem cerebral cortex and cerebellum of patients with autism the presence of activated microglia through the positive immunoreactivity for MHC class II and a unique pro-inflammatory cytokines profile, suggesting that these findings are indicative of a chronic inflammatory state in autism (Vargas et al., 2005). Later, another study was performed in order to analyze and quantify microglial cell abnormalities; microglial cells were identified using an anti Iba-1 antibody that labels both resting and activated microglial cells, whereas the soma volume and microglial density were analyzed through isotropic nucleator and optical fractionators (Morgan et al., 2010). This analysis revealed microglial cell activation in 5 of 13 patients including two cases under 6 years of age and showed that microglial cell abnormalities consisted of process retraction, extension of filopodia and soma enlargement. However, these morphological changes are not representative of an inflammatory event and it is not possible to address if these alterations are an index of microglial cell disturbance or consequence of the pathology (Morgan et al., 2010).

The neural circuits mature through various processes which include the formation of synapses, their pruning and the final maturation. These processes are necessary for a correct ratio between excitatory and inhibitory synapses (E/I). Indeed, in ASD patients, an unbalanced E/I ratio, especially an increase in this ratio, has been suggested as one main cause of classic ASD phenotypes (Rubenstein and Merzenich, 2003). Recently, interesting findings have linked impaired synaptic pruning during development to ASD (Tang et al., 2014). These results suggest that pruning of excitatory synapses could have been mitigated in ASD patients. However, in this study the role of microglia was not inquired. Other studies using a radiotracer for microglia conducted an analysis in ASD young adult patients. Microglial activation in brain regions, such as the anterior cingulate, orbitofrontal cortex and the cerebellum, whose dysfunction has been suggested in ASD, have been found (Suzuki K. et al., 2013). In living young (3–10 years old) ASD patients, microglial activation has also been evidenced by the pronounced release by microglia of pro-inflammatory chemokines such as monocyte chemoattractant protein-1 (MCP-1) in cerebrospinal fluid (Vargas et al., 2005).

In addition, previous works have shown that during pregnancy, maternal inflammation may lead to ASD similar phenotypes in both rodents and primates (Knuesel et al., 2014; Giovanoli et al., 2015). These works highlight the relationship between inherited risk for ASD and environmental exposures. Therefore, inherited risk for ASD might result in “primed” altered microglia that following exposure to external inflammatory factors or maternal inflammation might determine abnormal development of neural network as a consequence of excessive response of microglia (Knuesel et al., 2014). The presence of inherent abnormalities in microglia at epigenetic or genetic level and the resulting affected function in neurodevelopment may elucidate the contribution to autism risk that is believed to be accrued by interactions among environmental and intrinsic factors.

Moreover, trauma, adversity and stress during early life stages are main risk factors for the onset of depression (Heim and Nemeroff, 2001). Immune function is altered by early stress both at the time of exposure to the stressor and later in life. In this case, subsequent stressful challenges in addition to the immune system may alter endocrine, immune as well as behavioral responses (Ganguly and Brenhouse, 2015). Alterations of microglial functions as a result of early stress could be important to conferring predisposition to depression. In fact, prenatal stress, which promotes the subsequent depressive symptoms in the offspring, induces a long-lasting activation of hippocampal microglia together with an increased reactivity to systemic LPS administration (Diz-Chaves et al., 2012).

## Conclusions

A complex interplay between microglia and synaptic wiring occurs during normal neurodevelopment. In this context, in non-inflammatory conditions, microglia regulate both synapse formation and synapse plasticity, function and elimination (**Figure 4**). The role of microglia in synaptic pruning during postnatal development (a phase that overlaps with the beginning of many ASDs) appears to be particularly relevant. Microglia-mediated synaptic pruning during development has a critical role in sculpting neural circuit function and a primary deficit in microglia may contribute to circuit-level deficits across a range of neurodevelopmental disorders, including autism.

In neurodegenerative diseases, the role of microglia has always been considered for its classical inflammatory function, which could be, *per se*, both a cause and a consequence of neuronal damage. More recently, considering the growing understanding of the physiological roles of microglia in the CNS, research has focused on certain neuropsychiatric disorders which are not characterized by straightforward neuronal degeneration.

In ASD, the nature of the contribution of microglial dysregulation to pathophysiology has not been completely elucidated and may be subtle, relating to microglia non-inflammatory functions. More generally, in some neuropsychiatric disorders in which no marked neurodegeneration occurs, microglial dysregulation may be a hallmark of failure of neuroprotective functions, leading to vulnerability to neuroinflammation (**Figure 4**).

Previous works regarding immune regulation in autism have evidenced that destruction of adult neural circuitry by microglia may occur, probably through inflammatory microglia and as a consequence of external injuries (**Figure 4**). However, it is equally likely that in ASD the normal neurodevelopmental may be perturbed by microglia without inflammation. Until very recently, the involvement of microglia in the occurrence of ASDs was completely neglected and for this reason therapeutic strategies were almost exclusively designed to synaptic transmission and neuronal activity. It is likely that alterations of the normal microglial functions, specifically during the first years of life, could have serious implications for the development of the brain. A deficient and/or dysregulated immune response may significantly participate in the genesis of neurodevelopmental disorders that involve abnormal dendritic spine maintenance and dendritic arborization. Disorders conventionally considered as a consequence of isolated neural dysfunctions may in fact be significantly affected and even caused in some cases, by primary microglial dysfunction (microgliopathies).

Microglial activation is not an ‘all-or-none’ functional state, in fact multiple functional programs may confer specific adaptation to microglia for dealing different pathophysiological conditions. Microglia must be dynamic and plastic but also measured in their responses. Moreover, as keepers of the brain, microglia must also possess the capability to vigorously respond to pathological damages, when appropriate. Microglial activation and related inflammation has progressed as an adaptive process, allowing the elimination of pathological critical issue; however, in many pathological events, including Parkinson’s disease and AD, activated microglia become neurotoxic and damage glial cells and neurons.

Thus, the long standing view that microglia are, at best, inactive, unless provoked by pathogens, has been challenged. During development, microglia are necessary for the pruning of excess synapses and removal of apoptotic neurons. Thus, the dogma of microglia as a disease indicator is now obsolete, since experimental evidence shows that inactivity of microglia is dangerous as is their excessive activity. The CNS environment must favor an appropriate and specific response of microglia, rather than a weak and generic response, with the final goal of maintaining the CNS in health. The possibility is open that epigenetic factors (environmental risk) may exert their effect by modulating microglial functions in individuals with genetic alterations as well as in psychiatric illnesses. Genetic alterations lead to “primed” abnormal microglia that in individuals exposed to environment challenges may generate an abnormal microglial response that alters the normal neural network development (**Figure 4**).

## Future Perspective

There is still much to understand about the role of microglia during embryonic and postnatal development. It is reasonable to state that different microglial populations alternate in performing specific functions.

In this context, single-cell RNA-seq techniques have been used to highlight individual cell populations with typical expression pattern, in the mouse somatosensory cortex (Zeisel et al., 2015) and during embryonic development (Matcovitch-Natan et al., 2016). The results of Matcovitch-Natan et al. (2016) have clearly demonstrated the presence of three different microglial populations with distinct temporal patterns which alternate during the embryonic and postnatal development. Alterations of the temporal relationships between these populations may contribute to the development of neurobehavioral disorders.

Recently, RNA-seq techniques were flanked by high-dimensional cytometry and techniques that combine cytometry and mass spectrometry, the so-called mass cytometry (Bandura et al., 2009), commercially known as CyTOF (cytometry by time-of flight). CyTOF could allow the theoretical identification in single cells of more than 100 parameters if combined with already existing, equally performing data analysis programs (Mair et al., 2016). The development of computational methods that automatically identify populations in multidimensional flow cytometry data have been successfully used to identify the immune populations resident in the lung in mice deficient for GM-CSF receptor (Mair et al., 2016).

The high performance of the techniques outlined above complies with advancements in microglial mouse genetics. To date, the transgenic animal model that best suits the study of microglia in embryonic and postnatal development and in adults mice is the *Cx3cr1^Cre^* model (Cronk et al., 2015; Schafer et al., 2016). Thanks to the high and early expression of *Cx3cr1*, this model targets effectively microglia, but not cell populations continuously renewed, such as monocytes. Furthermore, the use of a Cre recombinase fused to the human estrogen receptor allowed the system to become inducible by tamoxifen and therefore usable by early embryonic stages until adulthood (*Cx3cr1^CreEr^*) (Wieghofer and Prinz, 2016). Finally, the recombination with loxP sites enables the inducible transcription of fluorescent reporter that allows following these cells from development through to adulthood. These systems have replaced previous models like *Csf1r*^Cre^ and *LyzM*^Cre^ models less suitable for this purpose (Wieghofer and Prinz, 2016).

The identification of specific microglial populations with well-defined transcriptional patterns will help to identify new microglial genes for even more specific transgenic models, which could shed light on the role of these cells in neurodevelopmental diseases.

## Author Contributions

CA wrote chapters “ROLE OF MICROGLIA IN NEURO DEVELOPMENTAL DISEASES,” “ROLE OF MICROGLIA IN NEURONAL DEVELOPMENT,” and “FUTURE PERSPECTIVE.” CM wrote the chapters “ORIGIN AND LOCALIZATION OF MICROGLIA” and “MOLECULAR MARKERS (PHENOTYPES).” IG wrote the chapter “MECHANISMS OF ACTION OF MICROGLIA.” RB wrote the chapter “ROLE OF MICROGLIA IN NEURONAL DEVELOPMENT-IMMUNOSURVEILLANCE.” RD wrote the INTRODUCTION and CONCLUSIONS.

## Conflict of Interest Statement

The authors declare that the research was conducted in the absence of any commercial or financial relationships that could be construed as a potential conflict of interest.

## Abbreviations

Aβ: amyloid β
AD: Alzheimer’s disease
Arg-1: Arginase-1
ASDs: autistic spectrum disorders
ATP: adenosine triphosphate
CCR2: C-C chemokine receptor type 2
CD: Cluster of Differentiation
CNS: central nervous system
CSF: colony stimulating factor
CSF-1R: colony stimulating factor-1 receptor
CX3CL1: C-X3-C motif ligand 1
CX3CR1: C-X3-C motif receptor 1
CyTOF: Cytometry by Time of Flight
DAMPs: Damage Associated Molecular Patters
DAP12: DNAX activation protein 12
dLGN: dorsal lateral geniculate nucleus
E/I: excitatory/inhibitory
EMPs: erythromyeloid precursors
ERK: extracellular signal regulated kinase
Fas: TNF receptor superfamily member 6
FasL: Fas ligand
Fc: fragment crystallizable
FTD: frontotemporal dementia
GAS6: growth arrest-specific protein 6
GFP: green fluorescent protein
GM-CSF: granulocyte-macrophage colony stimulating factors
Hdc: histidine decarboxylase
HDLS: Hereditary diffuse leukoencephalopathy with spheroids
HSC: hematopoietic stem cell
Iba-1: ionized calcium binding adaptor molecule-1
IFN: interferon
IGF-1: insulin-like growth factor 1
iNOS: inducible nitric oxide synthase
InsP3: inositol 1,4,5-trisphophate
IL: Interleukin
LPS: lipopolysaccharide
LRP: low-density lipoprotein receptor-related protein
MCP-1: monocyte chemoattractant protein-1
M-CSF: macrophage colony stimulating factor
MERTK: MER receptor tyrosine kinase
MFG-E8: milk fat globule EGF 8
MHC: Major Histocompatibility Complex
MIP: macrophage inflammatory protein
MyD88: myeloid differentiation factor 88
NF-κB: nuclear factor kappa-light-chain-enhancer of activated B cells
NGF: nerve growth factor
NHD: Nasu-Hakola disease
NO: nitric oxide
NPCs: neural precursor cells
PAMPs: pathogen-associated molecular patterns
PCD: programmed cell death
PS: phosphatidylserine
RAGE: receptor for advanced glycation end products
RGC: retinal ganglion cell
RNA-seq: RNA sequencing
RTT: Rett syndrome
SIGLECs: sialic acid-binding immunoglobulin-like lectins
TGF-β: transforming growth factor-β
TLRs: Toll Like Receptors
TNFs: tumor necrosis factors
TTREM2: Triggering receptor expressed on myeloid cells 2
TS: Tourette syndrome
UDP: uridine diphosphate
VNRs: vitronectin receptors
